# A serum Golgi Protein 73 (GP73)-based model for evaluating inflammation associated with drug-induced liver injury

**DOI:** 10.1016/j.clinsp.2026.101112

**Published:** 2026-09-06

**Authors:** Yamei Wei, Mingjie Yao, Mei Zhang, Shuhong Liu, Danli Yang, Ziyuan Ma, Hao Wu, Jingmin Zhao, Fengmin Lu

**Affiliations:** a Tianshui Center for Disease Control and Prevention, Qinzhou District, Tianshui, Gansu, China; b Department of Preventive Medicine, School of Medicine, Shihezi University, Shihezi, Xinjiang, China; c Department of Anatomy and Embryology, School of Basic Medical Sciences, Peking University Health Science Center, Beijing, China; d Hunan Provincial University Key Laboratory of the Fundamental and Clinical Research on Functional Nucleic Acid, Changsha, Hunan, China; e Department of Pathology and Hepatology, The Fifth Medical Center of PLA General Hospital, Beijing, China; f Department of Microbiology & Infectious Disease Center, School of Basic Medicine, Peking University Health Science Center, Beijing, PR China; g School of Medical Sciences, University of Sydney, Sydney, Australia

**Keywords:** Drug-induced liver injury, GP73, Liver inflammation, Liver fibrosis, Non-invasive diagnosis

## Abstract

**Objective:**

To evaluate the diagnostic utility of serum Golgi Protein-73 (GP73) in staging hepatic inflammatory activity and evaluating fibrosis in patients with Drug-Induced Liver Injury (DILI).

**Methods:**

A retrospective analysis was conducted on 130 patients with DILI who underwent liver biopsy. Patients were categorized according to inflammation grade (G0∼G1: n = 49, G2∼G3: n = 62, G4: n = 19) and fibrosis stages (S0∼S1: n = 24, S2∼S3: n = 45, S4: n = 14). Correlations between GP73 levels and markers of liver inflammation, fibrosis, and biochemical indicators were examined. Logistic regression and ROC curves evaluated diagnostic precision.

**Results:**

Serum GP73 levels exhibited a positive correlation with liver injury markers such as ALT, AST, TBIL, ALP, HA, PC-III, and APRI (*p* < 0.05), while demonstrating a negative correlation with ALB, CHE, PA, and FIB (*p* < 0.05). GP73 and ALB were recognized as independent risk factors for hepatic inflammation (*p* < 0.05). The integrated diagnostic efficacy of GP73 and ALB for moderate (G ≥ 2) and severe inflammation (G ≥ 3) produced AUCs of 0.860 and 0.945, respectively, surpassing those of ALT and AST (AUCs: 0.679, 0.716 and 0.762, 0.805, respectively). When ALT levels were normal, the combined AUCs of GP73 and ALB for diagnosing moderate (G ≥ 2) and severe inflammation (G ≥ 3) were 0.853 and 0.987, respectively. Furthermore, GP73 was identified as an independent predictor of fibrosis (*p* < 0.05), exhibiting AUCs of 0.707 and 0.856 for moderate and severe fibrosis, respectively, indicating enhanced diagnostic efficacy relative to FIB-4 and APRI.

**Conclusion:**

GP73 is significantly increased in the serum of patients experiencing drug-induced liver injury. GP73, whether utilized independently or alongside other diagnostic methods, exhibits significant potential for evaluating liver inflammation and fibrosis. Its efficacy in diagnosing liver inflammation exceeds its utility for fibrosis, potentially reducing the necessity for liver biopsy procedures.

## Highlights

• Serum GP73 levels are significantly elevated in patients with drug-induced liver injury.
• GP73 correlates strongly with histological grades of hepatic inflammation and fibrosis.
• GP73 combined with albumin improves diagnostic accuracy for DILI-related inflammation.
• GP73 outperforms ALT and AST in detecting moderate-to-severe inflammation.
• GP73 shows strong diagnostic value even in DILI patients with normal ALT levels.

## Introduction

Drug-Induced Liver Injury (DILI) refers to liver damage caused by pharmaceutical drugs, biological products, traditional or natural medicines (both prescription and over-the-counter), raw herbal materials, health supplements, dietary supplements, or their metabolic products, including excipients, contaminants, impurities, and other related substances.^1^

DILI is a prevalent cause of clinical liver disease, especially acute liver injury, and constitutes a significant factor in drug development failures and post-marketing withdrawals. As a result, it has attracted growing attention in recent years.^2^, ^3^, ^4^, ^5^ DILI has become a predominant cause of Acute Liver Failure (ALF) globally, with a rising incidence.^6^ The projected annual incidence of DILI in China is a minimum of 23.80 per 100,000 individuals.^7^ DILI represents 10% to 15% of all adverse drug reactions,^6^ with traditional Chinese medicine being a notable contributor, especially in China.^8^^,^^9^ The intricate pathogenesis and varied etiologies of DILI present significant clinical challenges.^10^^,^^11^ Prompt and precise diagnosis, coupled with evaluation of liver inflammation and fibrosis, is crucial for timely intervention and monitoring to avert complications such as Liver Cirrhosis (LC), end-stage liver disease, and Hepatocellular Carcinoma (HCC). At present, percutaneous liver biopsy is the definitive standard for diagnosing liver fibrosis. Nonetheless, its invasive characteristics are linked to possible complications and vulnerability to sampling errors.^12^ Furthermore, the lack of capability to dynamically assess the advancement of liver fibrosis restricts its extensive clinical utilization. Consequently, it is essential to identify noninvasive biochemical markers that can diagnose DILI-induced hepatitis and fibrosis to diminish reliance on liver biopsy.

Golgi Protein 73 (GP73) is a 73 kDa transmembrane protein situated on the cisternal membrane of the Golgi apparatus, also referred to as type II Golgi membrane protein or Golgi membrane protein I. This protein was initially identified by Kladney et al.^13^ in multinucleated giant cells originating from hepatocytes in patients with adult giant cell hepatitis. GP73 expression is heightened in hepatic malignancies^14^ and extracorporeal malignant tumors.^15^, ^16^, ^17^ However, have shown that serum GP73 levels are markedly increased in patients with advanced liver fibrosis and cirrhosis,^18^^,^^19^ which restricts its capacity to distinguish cirrhosis from liver cancer, consequently diminishing its diagnostic specificity for hepatocellular carcinoma.^20^^,^^21^ Previous research by our team and others have shown that serum GP73 possesses considerable diagnostic significance for compensated cirrhosis, as its expression escalates with the advancement of the disease from mild fibrosis to cirrhosis. The diagnostic potential has been noted across multiple etiologies, including Chronic Hepatitis B (CHB), Chronic Hepatitis C (CHC), Nonalcoholic Fatty Liver Disease (NAFLD), and autoimmune liver disease.^22^ Additionally, numerous studies have indicated a substantial correlation between GP73 levels and liver inflammation,^23^, ^24^, ^25^, ^26^, ^27^ with certain results proposing that GP73 is a more effective predictor of liver inflammation (Area Under the Curve ‒ AUC = 0.806) than fibrosis (AUC = 0.742).^24^ Our prior research demonstrated that the conjunction of GP73 and ALT improves the diagnostic precision for moderate to severe liver injury in patients with CHB. Thus, we hypothesize that serum GP73 may be a significant serological biomarker indicating the severity of hepatic inflammation and fibrosis in patients with DILI. This study sought to examine serum GP73 levels in patients with DILI and assess its diagnostic utility for liver tissue inflammation and fibrosis, aiming to offer new diagnostic markers and inform clinical management of DILI-related liver damage.

## Materials and methods

### Patients and methods

**Fig. 1:**
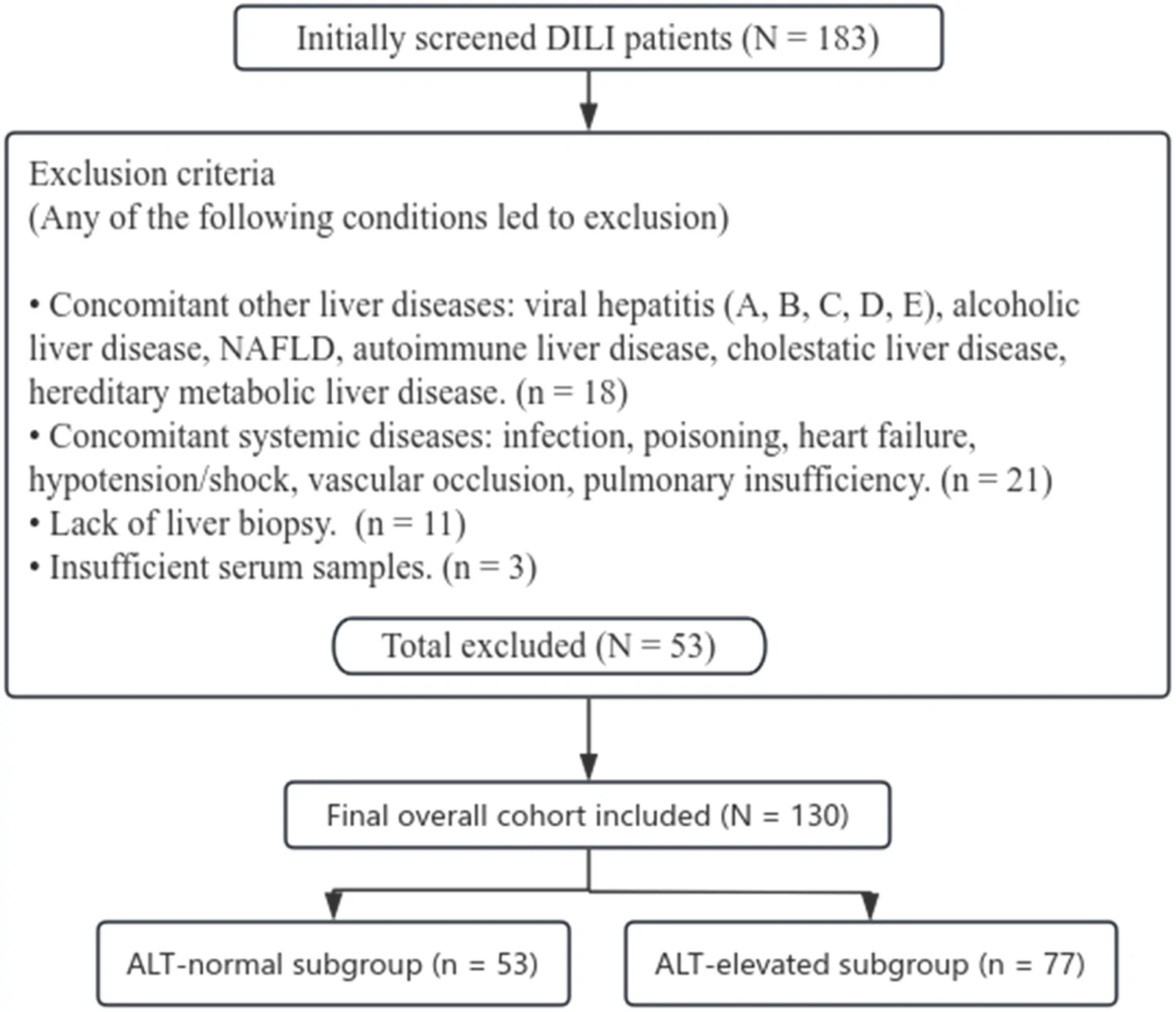
Flowchart of participant selection.

This study enrolled patients diagnosed with DILI at the Fifth Medical Center of the Chinese PLA General Hospital from June 2014 to December 2021. The diagnosis was determined according to the criteria specified in the Chinese Guidelines for the Diagnosis and Treatment of Drug-Induced Liver Injury, adhering to the principle of exclusion-based diagnosis.^1^ The study cohort consisted of 38 males and 92 females, aged 15 to 78 years, with a mean age of 49 years. The Roussel Uclaf Causality Assessment Method (RUCAM) scoring system was employed for causality assessment, augmented by expert opinions for a thorough evaluation.^1^ Patients were excluded if they had viral hepatitis (Hepatitis A, B, C, D, or E), alcoholic liver disease, Non-Alcoholic Fatty Liver Disease (NAFLD), autoimmune liver disease, cholestatic liver disease, or hereditary metabolic liver disease. Furthermore, individuals suffering from infections, poisoning, heart failure, hypotension or shock, vascular occlusion, or pulmonary insufficiency resulting in systemic hypoxic organ damage were excluded from the study^28^ (Fig. 1).

### Detection indicators

Fasting blood samples (5 mL) were obtained from patients the morning after admission, in addition to samples from healthy individuals undergoing routine physical examinations. Samples were centrifuged at 4000 rpm for 10 minutes, and the resultant serum was preserved at −80 °C for future analysis. Serum GP73 concentrations were measured utilizing a double-antibody sandwich Enzyme-Linked Immunosorbent Assay (ELISA) kit produced by Beijing Hotgen Biotech Co., Ltd., China, in accordance with the manufacturer's instructions. A fully automated biochemical analyzer was utilized to quantify serum aspartate Aminotransferase (AST), Alanine Aminotransferase (ALT), Total Bilirubin (TBIL), and Albumin (ALB). Liver fibrosis biomarkers, such as Serum Laminin (LN), type IV Collagen (IV-C), Hyaluronic Acid (HA), and Procollagen type III (PC-III), were identified utilizing a chemiluminescence immunoassay.

### Histological examination

Liver biopsy samples were obtained with the patient's informed consent. The specimens were preserved in buffered formalin, embedded in paraffin, and stained with Hematoxylin and Eosin (H&E) as well as Masson's trichrome stain. Histological assessment was conducted by seasoned pathologists who were unaware of the clinical information. Liver inflammation was assessed utilizing the Scheuer scoring system, with stages categorized as follows: stages G0-G1 indicate mild liver tissue inflammation, G2∼G3 indicate moderate liver tissue inflammation, and G4 indicates severe liver tissue inflammation. Among the DILI patients, 49 had G0∼G1 disease, 62 had G2∼G3 disease, and 19 had G4 disease. Liver fibrosis was evaluated utilizing the METAVIR scoring system, which categorizes it as follows: S0-S1 indicates mild liver fibrosis, S2∼S3 indicate moderate liver fibrosis, and S4 indicates severe liver fibrosis. Among the DILI patients, 64 were in the S0∼S1 stages, 50 were in the S2∼S3 stages, and 16 were in the S4 stage.

### Statistical methods

Statistical analysis was performed utilizing SPSS 26.0 software. Categorical variables were expressed as case counts and analyzed utilizing the Chi-Square (χ^2^) test. Normally distributed data were represented as mean ± Standard Deviation (χ±SD). Comparisons among multiple groups were conducted using one-way analysis of variance (ANOVA), followed by post hoc pairwise comparisons utilizing the Student-Newman-Keuls (SNK-q) test. Non-normally distributed data were presented as median (interquartile range, IQR; Q1–Q3). Comparisons between two groups were performed using the Mann-Whitney *U* test, and comparisons among multiple groups were performed using the Kruskal-Wallis H test. Spearman correlation analysis was performed to investigate the associations between serum GP73 levels and markers of liver inflammation and fibrosis. Logistic regression analysis was utilized to ascertain risk factors linked to liver inflammation and fibrosis. The diagnostic efficacy of serum GP73 in identifying liver inflammation and fibrosis was assessed through Receiver Operating Characteristic (ROC) curve analysis, with statistical significance established at p < 0.05.

## Results

### Patient characteristics

The classification of Drug-Induced Liver Injury (DILI) patients was conducted in accordance with the criteria outlined in the “Guidelines for Diagnosis and Treatment of Drug-Induced Liver Injury in China (2023 Edition)”: 1) Cholestatic type: R ≤ 2; 2) Mixed type: 2 < R < 5; and 3) Hepatocellular injury type: R ≥ 5., where R is calculated as (Actual ALT value/Upper Limit of Normal ALT) / (Actual ALP value/Upper Limit of Normal ALP). No substantial gender disparities were noted among the various DILI types; nonetheless, female patients exceeded male patients in total numbers. The mean age of patients with hepatocellular injury was significantly lower than that of patients with cholestatic and mixed injury types (*p* < 0.05). Serum concentrations of ALT, AST, TBIL, GGT, GP73, PLT, TT, APRI, DBIL, and TBA demonstrated a sequential elevation across cholestatic, mixed, and hepatocellular injury types, while PA levels exhibited a corresponding decline. The disparities were statistically significant among the various types of DILI (*p* < 0.05), as illustrated in Table 1. Subsequent pairwise comparisons utilizing the SNK-q test revealed statistically significant differences in ALT, AST, TBIL, GGT, DBIL, TT, APRI, TBA, and PA levels when differentiating hepatocellular injury from cholestatic and mixed types. Moreover, ALB and GP73 levels exhibited statistically significant variations between hepatocellular and cholestatic injury types (Table 1).

**Table 1:** Patient characteristics.

Variable	Overall	Cholestatic (n = 7)	Mixed type (n = 45)	Hepatocellular injury typ (n = 78)	H/χ^2^	p
Age (year)	49±11.74	51±17.89	53±9.63	47±11.82^a^^,^^b^	6.390	0.041
gender					0.829	0.661
Male, n (%)	38 (29.20)	1 (14.30)	14 (31.10)	23 (29.50)		
Female, n (%)	92 (70.80)	6 (85.70)	31 (68.90)	55 (70.50)		
ALT (U/L)	52.00 (25.75, 195.50)	10.00 (10.00, 12.00)	25.00 (17.00, 32.50)^a^	167.00 (54.50, 316.50)^a^^,^^b^	81.098	0.001^c^
AST (U/L)	58.00 (30.75, 241.75)	27.00 (20.00, 28.00)	38.00 (20.00, 52.00)	126.50 (56.75, 367.50)^a^^,^^b^	52.762	0.001^c^
ALP (U/L)	121.00 (87.00, 170.25)	110.00 (87.00, 164.00)	110.00 (79.50, 142.00)	128.00 (95.25, 175.25)	3.468	0.177
TBIL (μmol/L)	18.40 (12.80, 36.60)	12.10 (8.70, 16.20)	16.90 (11.75, 21.70)	20.90 (13.88, 111.35)^a^^,^^b^	13.979	0.001^c^
GGT (U/L)	83.50 (29.00, 172.50)	23.00 (17.00, 106.00)	47.00 (17.50, 112.50)	108.00 (49.50, 215.00)^a^^,^^b^	12.972	0.002^c^
INR	0.99 (0.93, 1.09)	0.95 (0.89,1.25)	0.98 (0.92, 1.09)	1.01 (0.94, 1.09)	1.252	0.535
GP73 (μg/L)	92.07 (55.17, 175.80)	55.53 (30.82, 168.40)	74.81 (47.21, 112.35)	128.30 (67.60, 206.65)^a^	12.808	0.002^c^
ALB (g/L)	37.00 (33.00, 40.00)	37.00 (29.00, 42.00)	37.00 (33.00, 40.00)	36.00 (32.75, 39.25)^a^	0.187	0.911
CHE (U/L)	54,920 (43,000, 7036)	5652 (2775, 8918)	5503 (4187, 7003)	5464 (4382, 7158)	0.464	0.793
PLT (10^9/L)	150.00 (112.75, 208.75)	138.00 (91.00, 194.00)	115.00 (71.00, 173.00)	163.50 (138.25, 225.25)	15.609	0.001^c^
FIB (g/L)	2.13 (1.56, 2.82)	2.85 (2.43,3.56)	2.29 (1.54, 2.85)	1.98 (1.55, 2.59)	4.873	0.087
TT (*sec*)	21.35 (19.75, 23.28)	18.00 (15.93, 21.13)	20.70 (19.35, 22.70)^a^	21.70 (20.40, 23.80)^a^^,^^b^	9.584	0.008^c^
FIB4	3.04 (1.61, 5.93)	2.29 (1.61, 4.58)	3.01 (1.69, 6.11)	3.41 (1.49, 5.93)	0.238	0.888
APRI	1.25 (0.51, 3.10)	0.36 (0.33, 0.77)	0.69 (0.40, 1.53)	2.45 (0.98, 4.97)^a^^,^^b^	32.401	0.001^c^
PR (%)	1.03 (0.90, 1.16)	1.05 (0.87, 1.23)	1.03 (0.88, 1.15)	1.04 (0.92, 1.17)	0.273	0.872
HA (mg/mL)	95.00 (52.00, 189.00)	95.89 (40.51, 206.00)	80.00 (48.00, 130.00)	121.50 (52.25, 189.50)	1.921	0.383
LN-IV (μg/mL)	76.00 (45.00, 156.00)	94.27 (31.51, 168.50)	68.00 (32.00, 110.00)	84.00 (50.25,170.75)	1.310	0.519
IV-C (μg/mL)	97.00 (44.00, 172.50)	133.00 (24.32, 329.44)	74.00 (44.00, 143.00)	103.00 (42.50, 273.00)	0.890	0.641
PC-III (ng/mL)	8.50(4.00,12.00)	9.50 (2.65, 12.32)	5.04 (3.13, 9.25)	10.00 (5.00, 14.00)	5.091	0.078
DBIL (mg/dL)	7.60(4.58,21.78)	4.60 (3.20, 8.50)	6.20 (4.00, 8.45)	9.20 (5.38, 71.18)^a^^,^^b^	15.703	0.001^c^
TBA (mg/dL)	20.00 (8.75, 72.00)	10.00 (4.00, 42.00)	15.00 (6.50, 37.40)	31.50 (10.75, 120.25)^a^^,^^b^	7.196	0.027^c^
PTA (%)	89.00 (77.90, 98.20)	100.60 (64.00, 110.50)	90.70 (77.15, 97.60)	88.60 (78.45, 97.60)	2.493	0.287
PA (mg/L)	132.50 (79.75, 187.25)	1940.00 (1050.00, 2030.00)	138.00 (1070.00, 2030.00)	110.00 (67.50, 159.75)^a^^,^^b^	12.268	0.002^a^

Note:.

a Indicates a significant difference compared to the cholestatic type, while.

b Indicates a significant difference compared to the mixed type.

c *p* ＜ 0.05 denotes statistical significance. ALP, Alkaline Phosphatase; ALT, Alanine Aminotransferase; AST, Aspartate Aminotransferase; GGT, Gamma-Glutamyl Transferase; PLT, Platelet Count; TBIL, Total Bilirubin; ALB, Albumin; INR, International Normalized Ratio; CHE, Cholinesterase; PLT, Platelet; FIB, Fibrinogen; TT, Thrombin Time; FIB4, Fibrosis-4 Index; APRI, AST-to-Platelet Ratio Index; PR, Prothrombin Ratio; HA, Hyaluronic Acid; LN-IV, Laminin; IV-C, type IV Collagen; PC-III, Procollagen type III; GP73, Golgi Protein 73; DBIL, Direct Bilirubin; TBA, Total Bile Acids; PTA, Prothrombin Activity; PA, Prealbumin.

### The evaluative value of serum GP73 and its combination with ALB for the severity of drug-induced liver injury

**Fig. 2:**
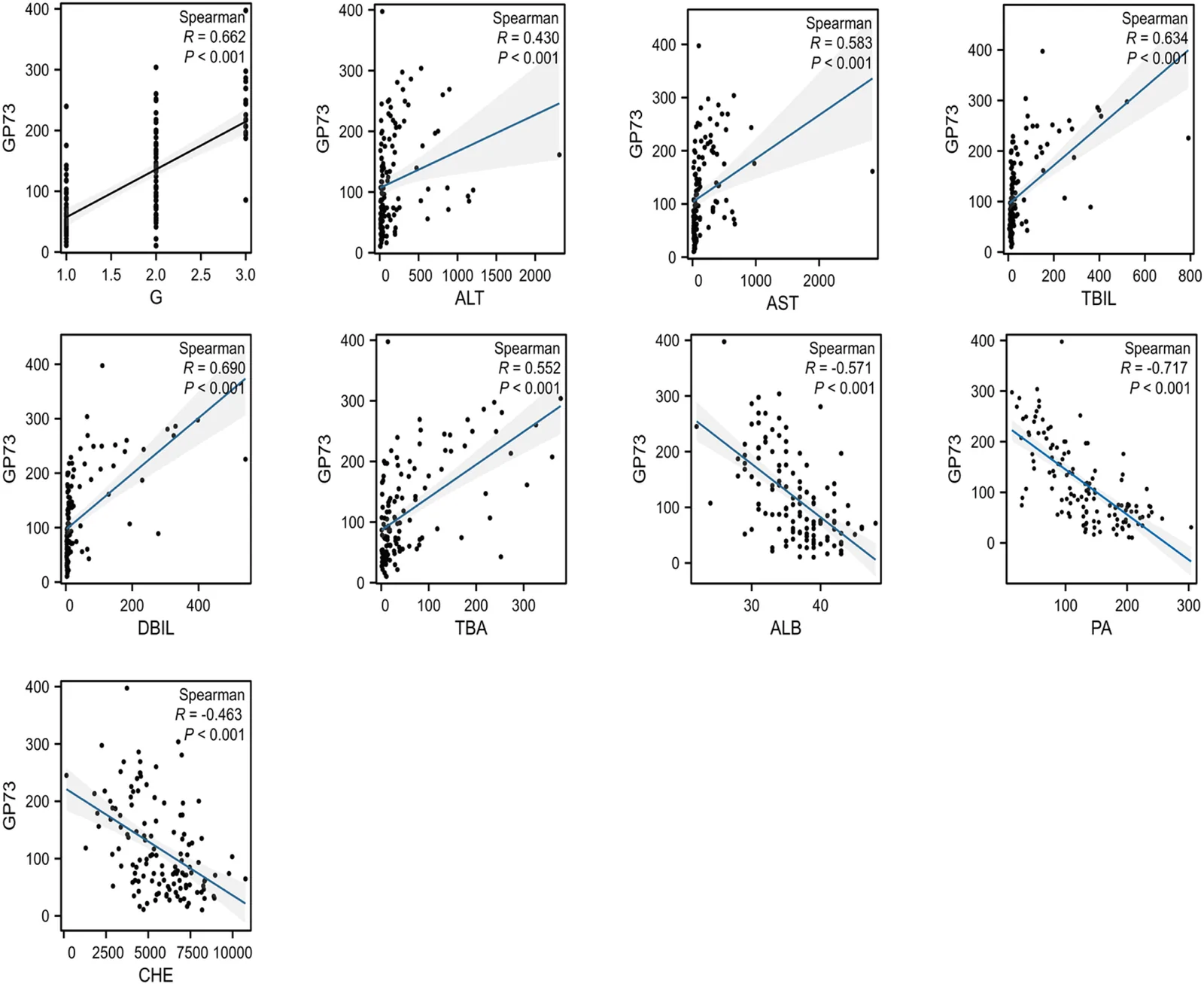
Correlation plot of the serum G, ALT, AST, TBIL, DBIL, TBA, ALB, PA, CHE and GP73 levels. Each plot displays the Spearman correlation coefficient (R) and p-value (P) in the upper right corner, with all p < 0.05. ALT, alanine aminotransferase; AST, Aspartate Aminotransferase; TBIL, Total Bilirubin; DBIL, Direct Bilirubin; TBA, Total Bile Acids; ALB, Albumin; PA, Prealbumin; CHE, Cholinesterase.

No significant differences in age, gender, ALP, GGT, and PLT levels were observed among the G0-G1, G2-G3, and G4 groups in patients with DILI. Serum levels of GP73, ALT, AST, TBIL, DBIL, and TBA demonstrated a progressive increase, whereas ALB, CHE, and PA levels exhibited a gradual decline. The disparities among the groups were statistically significant (*p* < 0.05), as indicated in Table 2. A logistic regression analysis was conducted to determine the risk factors affecting the severity of liver tissue inflammation in patients with Drug-Induced Liver Injury (DILI). The severity of liver tissue inflammation (G0-G1 = 0, G2-G4 = 1) was designated as the dependent variable, whereas continuous variables such as ALT, AST, GP73, ALB, CHE, PA, TBIL, DBIL, and TBA were considered independent variables. The findings demonstrated that GP73 and ALB were significant risk factors affecting the severity of liver tissue inflammation in patients with DILI (*p* < 0.05; Table 3). Spearman correlation analysis indicated a positive correlation between serum GP73 and inflammatory Grading (G), ALT, AST, TBIL, DBIL, and TBA (*r* = 0.662, 0.430, 0.583, 0.634, 0.690, 0.552; all *p* < 0.05). In contrast, serum GP73 exhibited a negative correlation with ALB, PA, and CHE (*r* = −0.571, −0.717, −0.463; all *p* < 0.05). Furthermore, ALB exhibited a positive correlation with CHE and PA (*r* = 0.747 for both; *p* < 0.05) and a negative correlation with AST, TBIL, DBIL, and TBA (*r* = −0.285, −0.456, −0.546, −0.449; all *p* < 0.05) (Fig. 2).

**Table 2:** Comparison of serum parameters at different inflammation levels (x̄±s).

Variable	Overall	G0∼G1 (n = 49)	G2∼G3 (n = 62)	G4 (n = 19)	F/H	p
Age (year)	49±11.74	50±11.01	49±12.64	45±10.15	3.464	0.177
Gender					0.674	0.714
Male, n (%)	38 (29.20)	16 (32.70)	16 (25.80)	6 (31.60)		
Female, n (%)	92 (70.80)	33 (67.30)	46 (74.20)	13 (68.40)		
ALT	52.00 (25.75, 195.50)	36.00 (21.00, 88.50)	54.00 (28.75, 205.00)	226.00 (139.00, 324.00)	18.819	0.001^a^
AST	58.00 (30.75, 241.75)	38.00 (26.50, 61.50)	64.50 (37.50, 279.75)	247.00 (137.00, 386.00)	26.431	0.001^a^
ALP	121.00 (87.00, 170.25)	110.00 (82.50, 154.50)	122.00 (83.75, 179.75)	144.00 (107.00, 170.00)	4.400	0.111
TBIL	18.40 (12.80, 36.60)	14.50 (9.95, 18.95)	18.8 5 (13.85, 28.58)	126.70 (33.20, 392.00)	33.735	0.001^a^
GGT	83.50 (29.00, 172.50)	57.00 (23.50, 135.50)	98.00 (24.75, 229.25)	100.00 (55.00, 215.00)	5.353	0.069
GP73	92.07(55.17,175.80)	53.42 (34.34, 87.13)	107.10 (73.90, 166.15)	225.60 (206.40, 269.20)	58.265	0.001^a^
ALB	37.00 (33.00, 40.00)	39.00 (36.00, 42.00)	36.00 (33.00, 39.00)	31.00 (30.00, 35.00)	33.277	0.001^a^
CHE	5492 (4300, 7036)	6256 (4721, 7350)	5400 (4300, 7027)	4397 (3738, 5377)	14.352	0.001^a^
PLT	150.00 (112.75, 208.75)	145.00 (116.50, 195.50)	148.00 (104.75, 212.25)	156.00 (125.00, 253.00)	1.671	0.434
DBil	7.60 (4.58, 21.78)	4.60 (3.40, 7.75)	8.05 (5.55, 16.33)	104.90 (24.20, 306.30)	40.468	0.001^a^
TBA	20.00 (8.75, 72.00)	11.00 (3.50, 33.00)	28.00 (11.75, 71.25)	135.00 (33.00, 192.00)	26.517	0.001^a^
PA	132.50 (79.75, 187.25)	156.00 (134.00, 203.00)	113.00 (85.00, 181.50)	57.00 (31.00, 76.00)	40.765	0.001^a^

The data are presented as n (%), mean ± standard deviation, and p-values were compared among the three groups.

a Indicates *p* < 0.05, indicating that the difference was statistically significant. ALP, Alkaline Phosphatase; ALT, Alanine Aminotransferase; AST, Aspartate Aminotransferase; GGT, Gamma-Glutamyl Transferase; PLT, Platelet Count; TBIL, Total Bilirubin; ALB, Albumin; CHE, Cholinesterase; PLT, Platelet; DBIL, Direct Bilirubin; TBA, Total Bile Acids; PA, Prealbumin; GP73, Golgi Protein-73.

**Table 3:** Logistic regression analysis of risk factors influencing liver tissue inflammation in patients with drug-induced liver injury.

Variable	β	SE	Wald χ^2^	OR	OR 95% CI	p
GP73	0.021	0.005	16.019	1.021	1.011‒1.031	0.001*
ALB	−0.230	0.084	7.582	0.794	0.674‒0.936	0.006*
constant	4.815	2.628	3.357	123.314		0.067

GP73, Golgi Protein-73; ALB, Albumin.

### The diagnostic value of serum GP73 for hepatic inflammation in patients with DILI

**Fig. 3:**
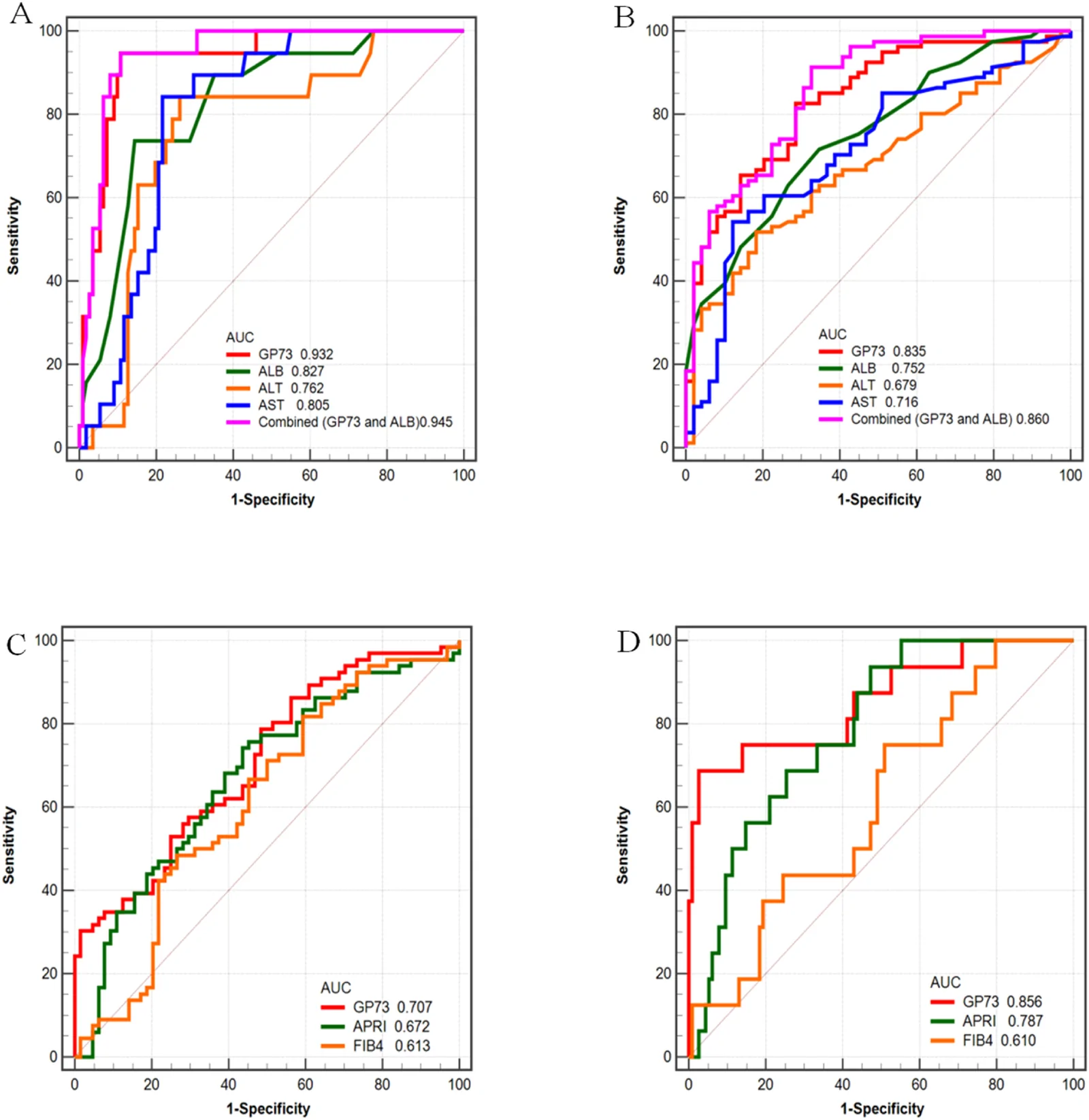
Receiver Operating Characteristic (ROC) curves for evaluating the diagnostic performance of serum GP73-based models in patients with Drug-Induced Liver Injury (DILI).(A) Diagnostic performance of GP73, Alanine Aminotransferase (ALT), Aspartate Aminotransferase (AST), Albumin (ALB), and GP73 combined with ALB for identifying moderate-to-severe hepatic inflammation (G ≥ 2). (B) Diagnostic performance of GP73, ALT, AST, ALB, and GP73 combined with ALB for identifying severe hepatic inflammation (G ≥ 3). (C) Comparison of GP73 with conventional fibrosis indices, including FIB-4 and APRI, for diagnosing moderate-to-severe liver fibrosis (S ≥ 2). (D) Comparison of GP73 with FIB-4 and APRI for diagnosing severe liver fibrosis (S ≥ 3). ALT, Alanine Aminotransferase; GP73, Golgi Protein-73; ALB, Albumin; AST, Aspartate Aminotransferase; Combined, GP73 and ALB; APRI, AST-to-platelet ratio index; FIB-4, Fibrosis-4 Index.

The diagnostic efficacy of serum GP73 for liver tissue inflammation in patients with DILI was evaluated using ROC curves. The AUCs for serum GP73 and ALB in diagnosing moderate or greater liver inflammation (G ≥ 2) in DILI patients were 0.835 and 0.752, respectively, with optimal cutoff values of 72.8 μg/L for GP73 and 37 g/L for ALB. The combined AUC for identifying moderate or greater inflammation utilizing both GP73 and ALB was 0.860, surpassing the individual AUCs of GP73 and ALB (*Z*-combined-GP73 = 1.146, *Z*-combined-ALB = 3.296, *p* = 0.2518, 0.0010). Furthermore, the integrated diagnosis surpassed the AUCs for ALT and AST in identifying moderate or greater liver inflammation (0.679 and 0.716, respectively) (*Z*-combined-ALT = 3.729, *Z*-combined-AST = 3.557, *p* = 0.0002, 0.0004). The AUCs for diagnosing severe liver inflammation (G ≥ 3) in patients with DILI were 0.932 for GP73 and 0.827 for ALB, with optimal cutoff values of 179 μg/L for GP73 and 32 g/L for ALB. The combined AUC for identifying severe inflammation was 0.945, suggesting superiority compared to the individual diagnoses of GP73 and ALB (*Z*-combined-GP73 = 1.154, *Z*-combined-ALB = 2.575, *p* = 0.2486, 0.0100). The combined diagnosis surpassed the AUCs for ALT and AST in identifying severe liver inflammation (0.762 and 0.805, respectively) (*Z*-combined-ALT = 3.073, *Z*-combined-AST = 3.282, *p* = 0.0021, 0.0010), (Table 4, Fig. 3). Additionally, we identified a cohort of individuals exhibiting normal Alanine Aminotransferase (ALT) levels (ALT < 40 U/L) to further evaluate the diagnostic efficacy of serum GP73, either independently or in conjunction with ALB, for the diagnosis of moderate and severe inflammation. The ideal cutoff thresholds for identifying moderate or greater inflammation (G ≥ 2) and severe inflammation (G ≥ 3) were 71.36 μg/L and 179 μg/L, respectively, with associated AUCs of 0.781 and 0.853 (*p* > 0.05) for moderate inflammation, and 0.987 for severe inflammation (*p* > 0.05). In addition, in the hepatocellular injury subgroup, the AUC values for GP73 combined with ALB in identifying moderate or greater and severe liver inflammation were 0.882 and 0.912, respectively. The sensitivity and specificity were elevated in this cohort, demonstrating that GP73 offers enhanced diagnostic utility for liver tissue inflammation in DILI patients, especially for identifying severe inflammation (Supplementary Tables 1 and 2, Fig. S1 and S2).

**Table 4:** Diagnostic performance of serum GP73, ALT, AST, FIB-4, and APRI for different histological lesions (inflammation and fibrosis) in patients with drug-induced liver injury.

Histological lesion type		Variable	AUC	AUC (95% CI)	Specificity (%)	Sensitivity (%)	Cutoff	PPV (%)	NPV (%)	Youden index
Inflammation Activity Grading	G ≥ 2 (n = 81)	GP73	0.835	0.760‒0.894	71.43	82.72	72.80	82.19	70.74	0.541
		ALB	0.752	0.669‒0.823	65.31	71.60	37.00	76.80	57.87	0.369
		ALT	0.679	0.591‒0.758	81.63	51.85	100.00	81.41	50.33	0.335
		AST	0.716	0.630‒0.792	87.76	54.32	92.00	87.14	53.69	0.421
		Combined	0.860	0.789‒0.915	67.35	91.36	0.41	81.82	82.02	0.587
	G ≥ 3 (n = 19)	GP73	0.932	0.874‒0.969	89.19	94.74	179.00	60.13	98.82	0.839
		ALB	0.827	0.751‒0.888	85.59	73.68	32.00	46.20	94.69	0.593
		ALT	0.762	0.679‒0.832	73.87	84.21	122.00	35.44	96.28	0.581
		AST	0.805	0.726‒0.869	78.38	84.21	121.00	40.26	96.51	0.626
		Combined	0.945	0.890‒0.977	89.19	94.74	0.93	60.13	98.82	0.839
Fibrosis Staging	S ≥ 2 (n = 66)	GP73	0.707	0.621‒0.783	51.56	78.79	71.36	62.36	69.01	0.304
		APRI	0.672	0.584‒0.752	56.25	74.24	0.94	63.64	67.42	0.305
		FIB4	0.613	0.523‒0.697	40.63	81.82	1.69	58.42	66.92	0.224
	S ≥ 3 (n = 16)	GP73	0.856	0.784 ‒0.912	97.37	68.75	229.00	75.56	95.70	0.661
		APRI	0.787	0.706 ‒0.854	52.63	93.75	1.14	20.90	98.20	0.464
		FIB4	0.610	0.521 ‒0.694	49.12	75.00	2.86	21.09	91.51	0.241

GP73, Golgi Protein 73; ALB, Albumin; ALT, Alanine Aminotransferase; AST, Aspartate Aminotransferase; combined, GP73 and ALB; APRI, AST-to-Platelet Ratio Index; FIB-4, Fibrosis-4 Index.

### Evaluation value of serum GP73 for the severity of fibrosis in DILI patients

**Fig. 4:**
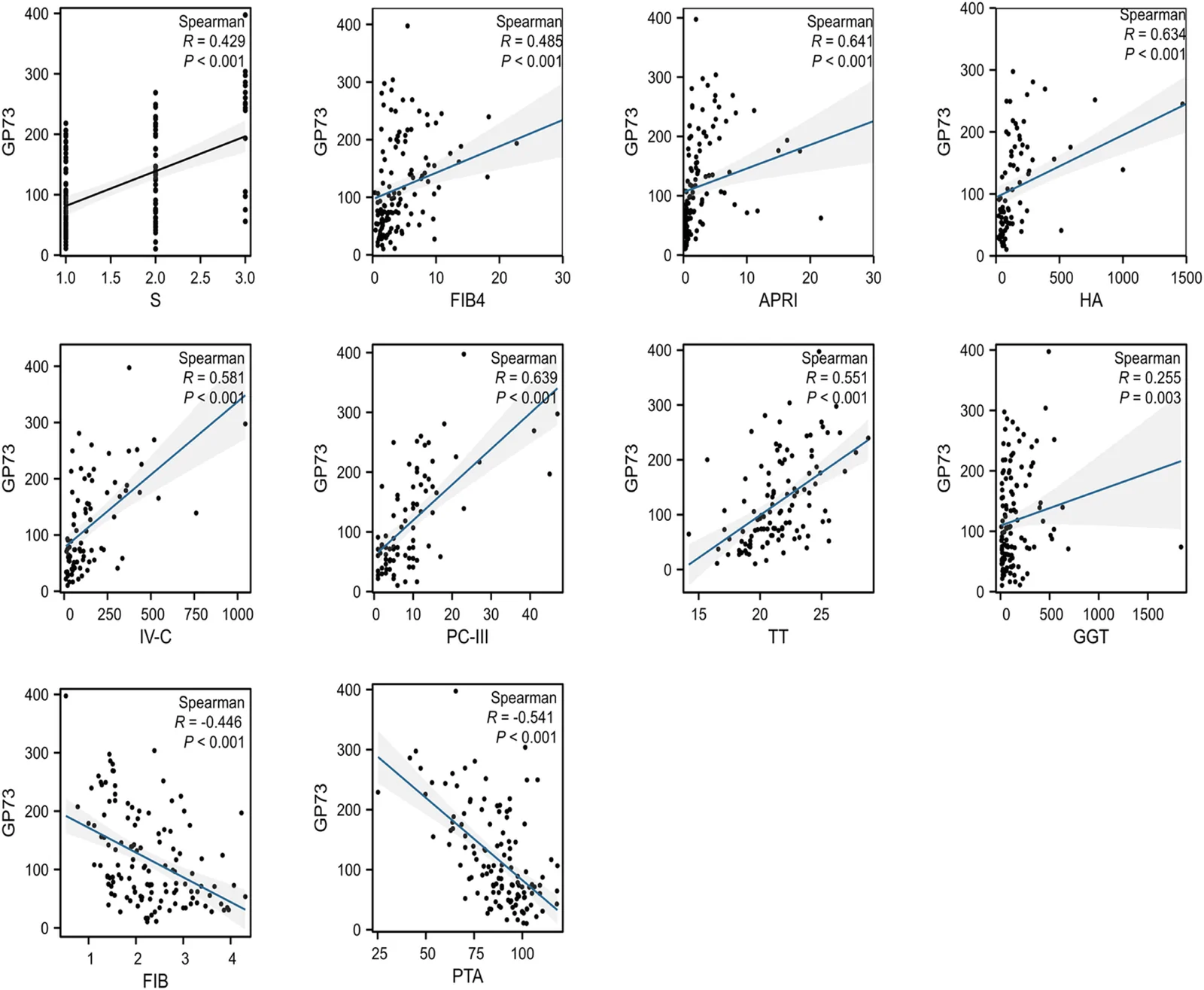
Correlation plot of the serum FIB-4, APRI, HA, IV-C, PC-III, TT, GGT, FIB, PTA and GP73 levels. Each plot displays the Spearman correlation coefficient (R) and p-value (P) in the upper right corner, with all p < 0.05. FIB-4, fibrosis-4 index; APRI, AST-to-platelet ratio index; HA, Hyaluronic Acid; IV-C, type IV Collagen; PC-III, Procollagen type III; TT, Thrombin Time; GGT, Gamma-Glutamyl Transferase; FIB, Fibrinogen; PTA, Prothrombin Activity; GP73, Golgi Protein 73.

Among DILI patients, no notable differences in age, gender, FIB4, LN-IV, PR, PLT, CHE, and PT levels were detected among the S0-S1, S2-S3, and S4 groups. Serum levels of GP73, HA, GGT, IV-C, PC-III, ALT, AST, DILI, TILI, TBA, and TT exhibited a progressive increase, while FIB, ALB, and PTA levels demonstrated a sequential decline, with statistically significant differences among groups (*p* < 0.05, Table 5). Logistic regression analysis, utilizing liver fibrosis severity (S0-S1 = 0, S2-S4 = 1) as the dependent variable, revealed that GP73 was an independent risk factor for liver fibrosis severity in DILI patients (*p* < 0.05, Table 6). Spearman correlation analysis revealed a positive correlation between serum GP73 and fibrosis Stage (S), FIB4 score, APRI, HA, IV-C, PC-III, TT, and GGT (*r* = 0.429, 0.485, 0.641, 0.634, 0.581, 0.639, 0.551, 0.255; all *p* < 0.05), and a negative correlation with FIB and PTA (*r* = −0.446, −0.541; both *p* < 0.05) (Fig. 4).

**Table 5:** Comparison of serum parameters at different liver fibrosis levels (x̄±s).

Variable	Overall	S0∼S1 (n = 24)	S2∼S3 (n = 45)	S4 (n = 14)	F/H	p
Age (year)	49±11.74	49±12.12	50±10.79	46±13.16	1.670	0.434
gender					2.997	0.223
Male, n (%)	38 (29.20)	20 (31.30)	11 (22.00)	7 (43.80)		
Female, n (%)	92 (70.80)	44 (68.80)	39 (78.00)	9 (56.30)		
FIB4	3.04 (1.61, 5.93)	2.45 (1.38, 4.34)	4.02 (1.84, 5.93)	3.44 (2.12, 7.18)	5.340	0.069
APRI	1.25 (0.51, 3.10)	0.77 (0.39, 2.14)	1.57 (0.60, 3.54)	4.39 (1.54, 7.46)	18.430	0.001*
GGT	83.50 (29.00, 172.50)	56.00 (22.25, 128.25)	100.00 (47.25, 200.75)	149.00 (64.00, 356.25)	10.321	0.006*
HA	95.00 (52.00, 189.00)	72.11 (46.00, 165.00)	110.50 (68.75, 192.00)	215.00 (81.25, 484.50)	8.187	0.017*
LN-IV	76.00 (45.00, 156.00)	61.58 (32.00, 140.25)	85.00 (56.00, 164.00)	95.50 (69.75, 542.50)	5.035	0.081
IV-C	97.00 (44.00, 172.50)	59.00 (30.15, 140.82)	123.00 (53.00, 215.00)	225.50 (88.25, 445.50)	10.400	0.006*
PC-III	8.50 (4.00, 12.00)	5.00 (3.00, 10.00)	10.00 (5.00, 13.00)	12.50 (8.00, 27.50)	11.881	0.003*
PR	1.03 (0.90, 1.16)	1.03 (0.89, 1.18)	1.01 (0.88, 1.11)	1.05 (1.01, 1.23)	2.931	0.231
GP73	92.07 (55.17, 175.80)	71.30 (39.75, 132.35)	107.40 (68.65, 175.80)	250.65 (127.05, 284.75)	27.724	0.001*
PLT	150.00 (112.75, 208.75)	150.00 (94.00, 200.50)	153.50 (112.75, 212.25)	155.50 (128.75, 231.25)	1.068	0.586
ALB	37.00 (33.00, 40.00)	37.00 (33.00, 40.75)	37.00 (34.00, 40.00)	33.50 (30.25, 35.75)	7.761	0.021*
CHE	5492.00 (4300.00, 7035.75)	5492.00 (4077.25, 7238.50)	6172.00 (4802.25, 7026.00)	4519.00 (4098.50, 6258.50)	3.953	0.139
ALT	52.00 (25.75, 195.50)	38.50 (22.00, 114.25)	52.50 (29.00, 195.75)	307.50 (127.00, 595.25)	16.586	0.005*
AST	58.00 (30.75, 241.75)	41.50 (27.25, 74.75)	66.00 (36.00, 284.25)	321.00 (107.25, 452.50)	12.942	0.002*
DBIL	7.60 (4.58, 21.78)	5.70 (3.93, 11.98)	7.65 (5.58, 16.25)	109.10 (28.20, 221.75)	20.468	0.001*
TBIL	18.40 (12.80, 36.60)	15.95 (11.15, 27.70)	18.40 (13.85, 25.25)	138.60 (45.50, 276.85)	18.186	0.001*
TBA	20.00 (8.75, 72.00)	17.50 (5.50, 55.25)	18.50 (8.00, 54.00)	82.00 (34.00, 232.50)	13.422	0.001*
PTA	89.00 (77.90, 98.20)	93.10 (81.70, 100.68)	88.80 (80.35, 94.60)	72.85 (64.23, 94.60)	8.208	0.017*
FIB	2.13 (1.56, 2.82)	2.29 (1.82, 2.96)	2.12 (1.61, 2.82)	1.51(1.28,2.18)	10.583	0.005*
TT	21.35 (19.75, 23.28)	20.65 (18.88, 22.70)	21.50 (20.53, 23.15)	22.60 (21.03, 25.20)	8.630	0.013*

FIB-4, Fibrosis-4 Index; APRI, AST-to-Platelet Ratio Index; GGT, Gamma-Glutamyl Transferase; HA, Hyaluronic Acid; LN-IV, Laminin; IV-C, type IV Collagen; PC-III, Procollagen type III; PR, Prothrombin Ratio; GP73, Golgi Protein-73; PLT, Platelet Count; ALB, Albumin; CHE, Cholinesterase; ALT, Alanine Aminotransferase; AST, Aspartate Aminotransferase; TBIL, Total Bilirubin; DBIL, Direct Bilirubin; TBA, Total Bile Acids; PTA, Prothrombin Activity; FIB, Fibrinogen; TT, Thrombin Time; PA, Prealbumin.

**Table 6:** Logistic regression analysis of risk factors influencing liver fibrosis in patients with drug-induced liver injury.

Variable	β	SE	Waldχ^2^	OR	OR 95%CI	p
GP73	0.008	0.003	6.097	1.008	1.002‒1.015	0.014
constant	−0.732	0.461	2.522	0.481		0.112

GP73, Golgi Protein 73.

### The diagnostic value of serum GP73 for hepatic fibrosis in patients with DILI

ROC curve analysis indicated that the AUC for serum GP73 in diagnosing moderate to severe fibrosis (S ≥ 2) was 0.707, with an optimal cutoff value of 71.36 μg/L, surpassing FIB4 (0.672) and APRI (0.613). In diagnosing severe fibrosis (S ≥ 3), GP73 exhibited an AUC of 0.856 with an optimal cutoff of 229 μg/L, surpassing FIB4 (0.787) and APRI (0.610) (Table 4, Fig. 3). In a cohort with normal ALT levels (ALT < 40 U/L), the GP73 threshold values for diagnosing moderate (S ≥ 2) and severe fibrosis (S ≥ 3) were 53.42 μg/L and 87.33 μg/L, respectively. In the hepatocellular injury subtype subgroup, the combined diagnostic performance of GP73 and ALB yielded AUC values of 0.736 and 0.853 for moderate-to-severe and severe liver fibrosis, respectively. GP73 demonstrated elevated sensitivity and specificity, especially in the diagnosis of severe fibrosis, thereby emphasizing its diagnostic significance (Supplementary Tables 3 and 4, Fig. S3 and S4).

## Discussion

Traditional serum biomarkers, including ALT, AST, ALP, and TBIL, are widely used for the diagnosis of liver injury. However, they have notable limitations. Although ALT is a sensitive indicator of hepatocellular injury, its specificity is limited, as elevations may also occur in conditions such as chronic hepatitis, muscle injury, or pregnancy.^29^ ALP is associated with cholestatic injury but lacks specificity and is often interpreted in conjunction with ALT to enhance diagnostic precision. TBIL reflects hepatic function; however, it can also be elevated in non-hepatic conditions, limiting its utility as an independent diagnostic marker. In contrast, GP73, a Golgi-associated protein elevated in various liver diseases, shows potential as a more specific biomarker for evaluating hepatic inflammation and fibrosis in DILI. Notably, its elevation differs mechanistically across DILI subtypes: in hepatocellular-type DILI, GP73 is released directly from damaged hepatocytes, while in cholestatic-type DILI, bile acid accumulation stimulates GP73 expression, though to a lesser degree.

One notable finding in our dataset (Table 1) is that patients classified as a cholestatic phenotype (n = 7) showed relatively low bilirubin levels (median TBIL 12.10 μmol/L), which is lower than typically observed in DILI. Clinical subtyping in this study was based on the R-value (calculated as (ALT/ULN)/(ALP/ULN)) according to the Chinese DILI guidelines rather than histopathological criteria. Under this definition, patients may be classified as cholestatic (R ≤ 2) even without marked hyperbilirubinemia, particularly in cases of mild injury. Consistently, this subgroup also demonstrated near-normal liver biochemistry, including ALT (median 10 U/L), AST (27 U/L), GGT (23 U/L), and ALP (110 U/L), indicating minimal overall injury severity. In addition, the timing of serum sampling may have contributed to these findings, as bilirubin typically increases later than aminotransferases in cholestatic DILI, and some patients may have been assessed at an early stage or during recovery. Therefore, the absence of significant hyperbilirubinemia does not invalidate the cholestatic classification. Nevertheless, this represents a limitation of the present study, and longitudinal sampling is recommended in future studies to better characterize bilirubin dynamics across DILI phenotypes.

Logistic regression identified GP73 and ALB as independent risk factors for hepatic inflammation, while GP73 also served as an independent predictor of fibrosis in DILI. Overall, GP73 levels significantly associated with hepatic inflammation (G), fibrosis (S), and related serological markers. These findings indicate that GP73 is a promising biomarker for assessing both hepatic fibrosis and inflammation in DILI patients. Superior diagnostic performance with an AUC of 0.932 and further improvement to 0.945 when combined with ALB, especially for severe inflammation (G ≥ 3), when compared to traditional biomarkers. Furthermore, the combination of GP73 and ALB demonstrated high diagnostic accuracy for both moderate (G ≥ 2) and severe (G ≥ 3) inflammation in patients with normal ALT levels, highlighting its potential usefulness even when liver enzyme levels stay within the normal range. In addition, GP73 showed superior performance for advanced fibrosis (S ≥ 3) compared with conventional fibrosis indices such as FIB-4 and APRI.

The diagnostic value of GP73 has been widely investigated in HBV, NAFLD, and HCV-related liver diseases. In HBV infection, GP73 has shown good diagnostic performance for moderate-to-severe inflammation, with AUC values ranging from 0.730,^19^^,^^24^^,^^30^, ^31^, ^32^, ^33^ and reported sensitivity and specificity of up to 97.3%. For fibrosis staging, AUC values ranging from 0.714 to 0.820^19^^,^^31^^,^^34^^,^^35^ for S ≥ 2 and 0.750 to 0.763 for S ≥ 3, demonstrating robust efficacy in cirrhosis detection with AUCs between 0.720 and 0.842.^18^^,^^19^^,^^32^^,^^35^^,^^36^ Moreover, the integration of GP73 with LSM significantly improves diagnostic precision, particularly in decompensated cirrhosis. In NAFLD, GP73 enhances conventional markers such as CK18-M30 and LSM, yielding AUCs of 0.741 and 0.891 for moderate and severe inflammation, respectively, and 0.857 for NASH.^26^ In HCV infection, its diagnostic performance is moderate (AUC: 0.717‒0.794),^37^ slightly inferior to established scores such as APRI and FIB-4. In Autoimmune Liver Disease (AILD), available evidence suggests moderate accuracy for fibrosis (AUC: 0.730‒0.855) and cirrhosis (AUC: 0.852‒0.880),^36^ although overlap with normal reference ranges remains a limitation.

Beyond viral and metabolic liver diseases, liver Ischemia-Reperfusion Injury (IRI) shares key pathological features with DILI, including oxidative stress, inflammation activation, and cholestatic injury.^38^ Bile acid accumulation further exacerbates hepatocellular damage, resembling mechanisms observed in cholestatic DILI.^39^ Importantly, GP73 is markedly upregulated in IRI and a positively correlates with inflammatory cytokines such as TNF-α and IL-6,^40^ supporting its role as a general marker of hepatic injury. The findings suggest that GP73 may serve as a cross-etiology biomarker for hepatic inflammation and injury, although further validation across different liver disease contexts is required.

In the present study, GP73 demonstrated superior diagnostic performance compared with conventional biomarkers such as ALT and AST, especially for severe inflammation (G ≥ 3). Its combination with ALB yielded further improvement (AUC = 0.945). Importantly, GP73 combined ALB maintained high diagnostic accuracy in patients with normal ALT levels, highlighting its potential value in clinically silent or enzyme-normal DILI cases. For fibrosis assessment, GP73 showed superior performance compared with FIB-4 and APRI, particularly in the diagnosis of severe fibrosis (S ≥ 3), achieving an AUC of 0.856 and a specificity of 97.37%. Subgroup analysis in hepatocellular-type DILI (n = 78). In this cohort, GP73 and GP73/ALB also demonstrated improved AUC values for diagnosing moderate-to-severe inflammation (G ≥ 2/G ≥ 3) and significant fibrosis (S ≥ 2). This enhanced performance may be attributed to GP73’s association with hepatocellular injury, reflecting its linkage to hepatocyte stress responses, Golgi apparatus dysfunction, and the activation of inflammatory signaling pathways. However, its performance for moderate fibrosis (S ≥ 2) was less robust, possibly due to overlap with normal reference ranges, suggesting limited sensitivity in early disease stages.

Overall, serum GP73 is significantly elevated in DILI and demonstrates strong diagnostic value for both liver inflammation and fibrosis. Its diagnostic performance appears superior for inflammation compared with fibrosis, consistent with prior reports. Compared with HBV, NAFLD, and HCV-related liver disease, GP73 shows higher AUC values in DILI-associated inflammation. This may reflect the acute and direct hepatocellular injury pattern of DILI, in contrast to the chronic and progressive remodeling processes seen in other liver diseases. Acute hepatocyte injury may lead to rapid GP73 release, thereby enhancing diagnostic sensitivity. Given the close interaction between inflammation and fibrosis progression through hepatic stellate cell activation and collagen deposition, early identification of inflammatory activity using GP73 may facilitate timely therapeutic decision-making, including drug withdrawal and monitoring strategies. As a non-invasive, easily measurable biomarker, GP73 represents a promising alternative to liver biopsy. This study has several limitations. First, it was a single-center retrospective study with a sample size of 130 patients, which was based on available cases rather than a priori sample size calculation, potentially limiting generalizability. Second, the lack of precise information on disease duration (time from DILI onset to sampling) may introduce chronicity-related bias, as variability in sampling time could affect both histological severity and GP73 levels. Third, incomplete documentation of causative drugs limited analysis of drug-specific hepatotoxic mechanisms. Future multicenter prospective studies with larger cohorts are needed. Standardized recording of onset time, longitudinal biomarker sampling, and detailed medication exposure will be essential. Integration of GP73 with LSM and imaging-based modalities may further improve diagnostic accuracy and clinical applicability.

## Conclusion

In summary, our research validates GP73 as a reliable biomarker for evaluating hepatic inflammation and fibrosis in patients with DILI. It exhibits greater diagnostic efficacy for inflammation than for fibrosis, with markedly enhanced performance relative to conventional markers. The mechanistic parallels between DILI and IRI reinforce GP73′s potential as a universal biomarker for hepatic damage. Future research must concentrate on corroborating GP73′s function in various liver diseases and optimizing its diagnostic thresholds to improve clinical applicability and patient management approaches.

## Data availability

The data used to support the findings of this study have not been made available because of privacy concerns.

## Ethics approval and consent to participate

This study was approved by the Ethics Committee (Approval No.: IRB00001052–19,081). Written informed consent was obtained from all participants. All procedures involving human participants adhered to the ethical standards of the institutional and/or national research committee, as well as the 1964 Helsinki Declaration and its subsequent amendments or comparable ethical standards.

## Patient consent for publication

Not applicable.

## Authors’ contributions

Wei YM and Yao MJ contributed equally to this work. The study concept and design (Wei YM, Yao MJ), acquisition of data (Yao MJ, Liu SH), analysis and interpretation of the data (Wei YM, Wu H), drafting of the manuscript (Wei YM, Wu H, Ma ZY), and critical revision of the manuscript for important intellectual content (Yao MJ, Lu FM, Zhao JM, Yang DL, Zhang M) were performed. Yao MJ and Liu SH confirm the authenticity of all raw data. All authors have made significant contributions to this study and have approved the final manuscript.

## Funding

This work was supported by the 10.13039/501100001809National Natural Science Foundation of China Grant (No. 82272433, 82203797) and the fund of Fujian Provincial Key Laboratory of Hepatic Drug Research (No. 2022-YF-0050).

## Declaration of competing interest

The authors declare no conflicts of interest.

## Appendix Supplementary materials

Image, application 1
