# Supplementary material for: A serum Golgi Protein 73 (GP73)-based model for evaluating inflammation associated with drug-induced liver injury
Source: Clinics (Sao Paulo). 2026 Sep 6;81:101112. doi: 10.1016/j.clinsp.2026.101112 (PMC13572763; doi:10.1016/j.clinsp.2026.101112)
Supplement: Supplementary file 1 [file mmc1.docx]

**CLINICS-D-26-00108_Supplementary Materials**

**Supplementary Table**

**Supplementary Table 1** Diagnostic value of serum GP73 for liver tissue inflammation in patients with drug-induced liver injury when ALT is normal.

| **Inflammation Activity Grading** | **Variable** | **AUC** | **AUC (95% CI)** | **Specificity (%)** | **Sensitivity (%)** | **Cutoff** | **PPV (%)** | **NPV (%)** | **Youden index** |
| --- | --- | --- | --- | --- | --- | --- | --- | --- | --- |
| G ≥ 2 (n = 27) | GP73 | 0.781 | 0.646‒0.883 | 73.08 | 74.07 | 71.36 | 73.27 | 73.74 | 0.472 |
|  | ALB | 0.769 | 0.633‒0.874 | 92.31 | 48.15 | 33.00 | 85.71 | 63.89 | 0.400 |
|  | ALT | 0.531 | 0.389‒0.669 | 73.08 | 40.74 | 27.00 | 59.70 | 54.89 | 0.138 |
|  | AST | 0.587 | 0.443‒0.720 | 73.08 | 55.56 | 33.00 | 67.07 | 61.86 | 0.286 |
|  | Combined | 0.853 | 0.729‒0.935 | 69.23 | 88.89 | 0.41 | 73.95 | 85.19 | 0.581 |
| G ≥ 3 (n =3) | GP73 | 0.987 | 0.908‒1.000 | 96.00 | 100.00 | 179.00 | 61.48 | 100.00 | 0.960 |
|  | ALB | 0.847 | 0.721‒0.931 | 98.00 | 66.67 | 26.00 | 67.81 | 97.83 | 0.647 |
|  | ALT | 0.663 | 0.520‒0.787 | 54.00 | 66.67 | 23.00 | 8.39 | 96.14 | 0.200 |
|  | AST | 0.867 | 0.745‒0.944 | 76.00 | 66.67 | 43.00 | 14.93 | 97.22 | 0.420 |
|  | Combined | 0.987 | 0.908‒1.000 | 96.00 | 100.00 | 0.93 | 61.48 | 100.00 | 0.960 |

GP73, Golgi Protein 73; ALB, Albumin; ALT, Alanine Aminotransferase; AST, Aspartate Aminotransferase; combined, GP73 and ALB.

**Supplementary Table 2** Diagnostic value of serum GP73 for liver tissue inflammation in patients with drug-induced liver injury when hepatocellular injury.

| **Inflammation Activity Grading** | **Variable** | **AUC** | **AUC (95% CI)** | **Specificity (%)** | **Sensitivity (%)** | **Cutoff** | **PPV (%)** | **NPV (%)** | **Youden index** |
| --- | --- | --- | --- | --- | --- | --- | --- | --- | --- |
| G ≥ 2 (n = 56) | GP73 | 0.881 | 0.788‒0.944 | 72.73 | 87.50 | 72.78 | 89.20 | 68.60 | 0.602 |
|  | ALB | 0.775 | 0.667‒0.862 | 81.82 | 66.07 | 36.00 | 90.33 | 48.40 | 0.478 |
|  | ALT | 0.702 | 0.588‒0.800 | 90.91 | 48.21 | 204.0 | 93.17 | 40.57 | 0.390 |
|  | AST | 0.781 | 0.673‒0.867 | 77.27 | 82.14 | 74.00 | 90.28 | 62.72 | 0.594 |
|  | Combined | 0.882 | 0.789‒0.944 | 95.45 | 67.86 | 0.69 | 97.46 | 53.58 | 0.633 |
| G ≥ 3 (n = 17) | GP73 | 0.900 | 0.811‒0.956 | 81.00 | 94.12 | 176.10 | 58.28 | 97.99 | 0.760 |
|  | ALB | 0.871 | 0.776‒0.936 | 90.16 | 76.47 | 32.00 | 68.67 | 93.14 | 0.666 |
|  | ALT | 0.687 | 0.572‒0.787 | 52.46 | 94.12 | 122.00 | 35.84 | 96.94 | 0.466 |
|  | AST | 0.721 | 0.608‒0.817 | 62.30 | 94.12 | 121.00 | 41.32 | 97.41 | 0.564 |
|  | combined | 0.912 | 0.826‒0.964 | 85.25 | 94.12 | 0.94 | 64.27 | 98.10 | 0.794 |

GP73, Golgi Protein-73; ALB, Albumin; ALT, Alanine Aminotransferase; AST, Aspartate Aminotransferase; combined, GP73 and ALB.

**Supplementary Table 3** Diagnostic value of serum GP73 for liver fibrosis in patients with drug-induced liver injury when ALT is normal and comparison with the diagnostic value of the FIB4 score and APRI for liver fibrosis in patients with drug-induced liver injury.

| **Fibrosis Staging** | **Variable** | **AUC** | **AUC (95% CI)** | **Specificity (%)** | **Sensitivity (%)** | **Cutoff** | **PPV (%)** | **NPV (%)** | **Youden index** |
| --- | --- | --- | --- | --- | --- | --- | --- | --- | --- |
| S ≥ 2 (n = 20) | GP73 | 0.573 | 0.429‒0.708 | 45.45 | 65.00 | 53.42 | 42.01 | 67.72 | 0.170 |
|  | APRI | 0.533 | 0.391‒0.672 | 39.39 | 75.00 | 0.41 | 42.97 | 71.79 | 0.144 |
|  | FIB4 | 0.532 | 0.390‒0.670 | 66.67 | 50.00 | 3.76 | 47.41 | 68.29 | 0.167 |
| S ≥ 3 (n = 2) | GP73 | 0.824 | 0.694‒0.915 | 64.71 | 100.00 | 87.33 | 10.37 | 100 | 0.647 |
|  | APRI | 0.755 | 0.617‒0.863 | 64.71 | 50.00 | 0.93 | 5.47 | 96.85 | 0.140 |
|  | FIB4 | 0.618 | 0.474‒0.748 | 72.55 | 50.00 | 5.15 | 6.93 | 97.19 | 0.220 |

GP73, Golgi protein-73; APRI, AST-to-Platelet Ratio Index; FIB-4, Fibrosis-4 Index.

**Supplementary Table 4** Diagnostic value of serum GP73 for liver fibrosis in patients with drug-induced liver injury when hepatocellular injury and comparison with the diagnostic value of the FIB4 score and APRI for liver fibrosis in patients with drug-induced liver injury.

| **Fibrosis Staging** | **Variable** | **AUC** | **AUC (95% CI)** | **Specificity (%)** | **Sensitivity (%)** | **Cutoff** | **PPV (%)** | **NPV (%)** | **Youden index** |
| --- | --- | --- | --- | --- | --- | --- | --- | --- | --- |
| S ≥ 2 (n = 47) | GP73 | 0.736 | 0.624‒0.829 | 96.77 | 38.30 | 206.40 | 95.09 | 48.97 | 0.351 |
|  | APRI | 0.693 | 0.578‒0.792 | 58.06 | 76.60 | 1.31 | 74.87 | 60.33 | 0.347 |
|  | FIB4 | 0.693 | 0.579‒0.793 | 45.16 | 91.49 | 1.45 | 73.13 | 76.46 | 0.367 |
| S ≥ 3 (n = 15) | GP73 | 0.853 | 0.755‒0.923 | 96.83 | 73.33 | 229.00 | 84.31 | 93.91 | 0.702 |
|  | APRI | 0.717 | 0.604‒0.814 | 36.51 | 100 | 1.14 | 26.97 | 100 | 0.365 |
|  | FIB4 | 0.630 | 0.513‒0.736 | 25.40 | 100 | 1.39 | 23.97 | 100 | 0.254 |

GP73, Golgi Protein-73; APRI, AST-to-Platelet Ratio Index; FIB-4, Fibrosis-4 Index.

**Figure S1** ALT < 40 U/L, ROC curves of GP73 and ALB for diagnosing liver tissue inflammation: (A) Moderate to severe inflammation and necrosis (G ≥ 2); (B) Severe to severe inflammation (G ≥ 3). ALT, Alanine Aminotransferase; GP73, Golgi Protein-73; ALB, Albumin; AST, Aspartate Aminotransferase; Combined, GP73 and ALB.

**Figure S2** Hepatocellular injury subgroup, ROC curves of GP73 and ALB for diagnosing liver tissue inflammation: (A) Moderate to severe inflammation and necrosis (G ≥ 2); (B) Severe to severe inflammation (G ≥ 3). ALT, Alanine Aminotransferase; GP73, Golgi Protein-73; ALB, Albumin; AST, Aspartate Aminotransferase; Combined, GP73 and ALB.

**Figure S3** ALT < 40 U/L, ROC curves of GP73 for diagnosing liver fibrosis and comparison with FIB4 and APRI: (A) Moderate to severe liver fibrosis (S ≥ 2); (B) Severe liver fibrosis (S ≥ 3).

**Figure S4** Hepatocellular injury subgroup, ROC curves of GP73 for diagnosing liver fibrosis and comparison with FIB4 and APRI: (A) Moderate to severe liver fibrosis (S≥2); (B) Severe liver fibrosis (S≥3).
